# Time-Course Global Expression Profiles of *Chlamydomonas reinhardtii* during Photo-Biological H_2_ Production

**DOI:** 10.1371/journal.pone.0029364

**Published:** 2011-12-29

**Authors:** Anh Vu Nguyen, Joerg Toepel, Steven Burgess, Andreas Uhmeyer, Olga Blifernez, Anja Doebbe, Ben Hankamer, Peter Nixon, Lutz Wobbe, Olaf Kruse

**Affiliations:** 1 Department of Algae Biotechnology and Bioenergy, Faculty of Biology, Bielefeld University, Bielefeld, Germany; 2 Division of Biology, Faculty of Natural Sciences, Imperial College London, London, United Kingdom; 3 Institute for Molecular Bioscience, The University of Queensland, St Lucia2, Australia; United States Department of Agriculture - Agricultural Research Service, United States of America

## Abstract

We used a microarray study in order to compare the time course expression profiles of two *Chlamydomonas reinhardtii* strains, namely the high H_2_ producing mutant *stm6glc4* and its parental WT strain during H_2_ production induced by sulfur starvation. Major cellular reorganizations in photosynthetic apparatus, sulfur and carbon metabolism upon H_2_ production were confirmed as common to both strains. More importantly, our results pointed out factors which lead to the higher H_2_ production in the mutant including a higher starch accumulation in the aerobic phase and a lower competition between the H_2_ase pathway and alternative electron sinks within the H_2_ production phase. Key candidate genes of interest with differential expression pattern include *LHCSR3*, essential for efficient energy quenching (qE). The reduced LHCSR3 protein expression in mutant *stm6glc4* could be closely related to the high-light sensitive phenotype. H_2_ measurements carried out with the *LHCSR3* knock-out mutant *npq4* however clearly demonstrated that a complete loss of this protein has almost no impact on H_2_ yields under moderate light conditions. The nuclear gene disrupted in the high H_2_ producing mutant *stm6glc4* encodes for the mitochondrial transcription termination factor (mTERF) MOC1, whose expression strongly increases during –S-induced H_2_ production in WT strains. Studies under phototrophic high-light conditions demonstrated that the presence of functional MOC1 is a prerequisite for proper LHCSR3 expression. Furthermore knock-down of *MOC1* in a WT strain was shown to improve the total H_2_ yield significantly suggesting that this strategy could be applied to further enhance H_2_ production in other strains already displaying a high H_2_ production capacity. By combining our array data with previously published metabolomics data we can now explain some of the phenotypic characteristics which lead to an elevated H_2_ production in *stm6glc4*.

## Introduction

The sulfur starvation method [1] for continuous hydrogen production in the green alga *Chlamydomonas reinhardtii* has received a lot of attention in the last decade as it improved the obtainable hydrogen yield significantly [2]. Under anaerobic conditions, *C. reinhardtii* and a number of other photosynthetic microorganisms can produce H_2_ via hydrogenase enzymes [3]. The production of H_2_ re-oxidizes reduced ferredoxin thereby maintaining essential ATP production [4]. Under illuminated conditions, H_2_ production is normally short-lived due to the inhibitory effects of O_2_ produced by photosynthesis on hydrogenase expression and activity [5]. By depriving the algae of sulfur, the photosynthesis to respiration ratio is decreased to less than one, effectively removing the dissolved O_2_ in the sealed culture yielding conditions supportive of anaerobic H_2_ production [1]. During S-deprived H_2_ production, major reorganizations of cellular structures and metabolic pathways occur within *C. reinhardtii* to aid survival [6]–[11]. First, the cell is reported to switch into the enhanced S acquisition/assimilation mode and as a result the transcript abundance of responsible enzymes greatly increases [6], [9]. In parallel, photosynthesis is down-regulated in response to the lower assimilation capacity. The decrease in photosynthesis was observed widely in light harvesting proteins, reaction centers and components of the electron transport chain as well as in components of the Calvin cycle when transcript [6] or protein levels [7], [8] of respective genes were analyzed. Enhanced protein degradation was also evident while certain proteins with lower S content are proposed to replace the function of their counterparts [6], [9]. Induction of anaerobiosis through sulfur depletion also triggers starch and lipid accumulation as shown in metabolomic studies on S-deprived H_2_ production [10], [11]. Upon the establishment of anaerobiosis due to the continuous net O_2_ consumption, additional sets of changes occur. Aerobic metabolic processes including citric acid cycle and oxidative phosphorylation are suppressed and replaced by fermentative pathways including H_2_ production [6], [10]–[12]. Due to the complexity of S-starvation induced H_2_ production, many factors have influences on the final H_2_ productivity. Reduced carbon sources such as starch or acetate are required for H_2_ase expression as their consumption is needed to drive *C. reinhardtii* cultures into anaerobiosis before H_2_ production can occur [12]–[15]. The starting pH was shown to have strong influences on H_2_ production with an optimum pH of 7.3 [16]. Availability of alternative electron sinks like carbon fixation as well as the activity of reductive enzymatic reactions coupled to ferredoxin also determine H_2_ productivity since both can reduce electron flow through the H_2_ase pathway [17]–[19]. The duration of the aerobic phase also determines the onset of H_2_ production and also affects how much energy is stored, consumed or made available to HYDA. Previous observations suggest that a short aerobic phase is desirable for S-deprived H_2_ production since it reduces the consumption of energy storage compounds such as starch and lipids. However, eliminating the aerobic phase altogether led to lower productivities [17], presumably due to the lack of the essential energy accumulation phase associated with fully functional oxygenic photosynthesis. As has been shown recently, enhanced oxygen consumption by introduction of leghemoglobin and ferrochelatase into *Chlamydomonas* is a means to improve hydrogen production [20].

S-starvation induced H_2_ production has been studied using different high-throughput technologies including transcriptomics [6], proteomics [7] and metabolomics [10]–[11]. Together with other studies on individual aspects of H_2_ production, these studies have contributed to the increasingly complete picture of the H_2_ production mechanism in *C. reinhardtii* with the identification of important target genes and pathways which can be used for future improvements. In this study we took one step further, using microarray to analyze and compare the time-course global expression profiles of two *C. reinhardtii* strains under S-deprived H_2_ production. A time-resolved transcriptome study takes into account that the H_2_ production process is composed of two main phases. In phase 1 anaerobiosis is established thereby activating the H_2_ase pathway and in phase 2 H_2_ production is sustained by the consumption of intracellular energy stores generating electrons which feed into the H_2_ase pathway [11]. In both phases regulative mechanisms occur, which represent potential targets for genetic engineering approaches designed to improve photobiological H_2_ production. An improved knowledge about the temporal aspects of transcriptome changes required to trigger and sustain H_2_ production in *C. reinhardtii* should enable more targeted genetic engineering strategies (e.g. by using inducible overexpression or knock-down systems). In addition they provide further insights into the transcriptomic differences between the high H_2_ producing strain *stm6glc4* [21], [22] and a wild type strain in order to understand why this mutant produces higher amounts of H_2_ within a given time.

## Results and Discussion

### The acclimatory response to sulfur deprivation differs largely between *stm6glc4* and wild type

**Figure 1:**
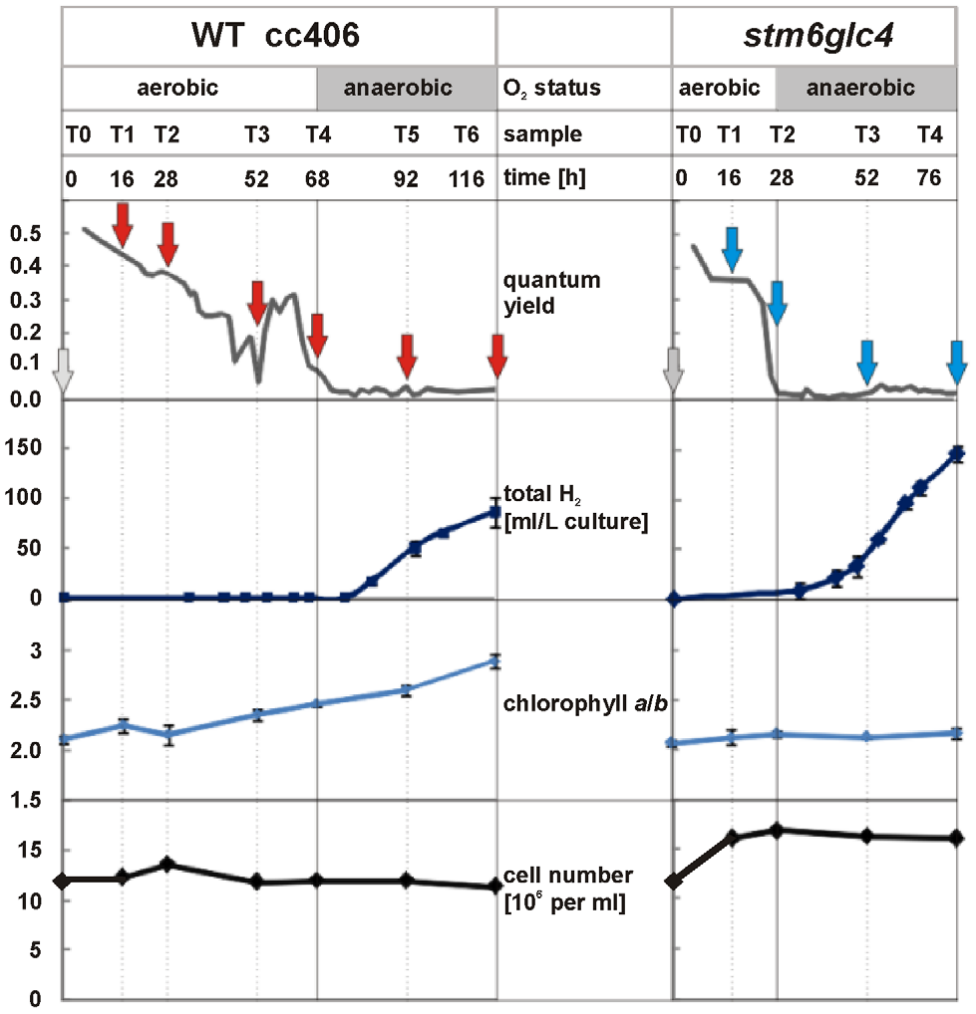
Changes in quantum yield, total H_2_, chlorophyll a/b ratio and cell number of the two strains *stm6glc4* and WT cc406 during the course of S-deprived H_2_ production. Arrows below the different time points indicate where samples were taken for microarray analysis.

In this study, we took samples at various time points during the course of S-deprived H_2_ production. Samples were simultaneously taken for parallel metabolome analyses reported in a previous study [11]. Figure 1 depicts the sampling points and how quantum yield (ΦPSII), total H_2_ yield, chlorophyll *a*/*b* ratio and cell number changed in the two strains during the course of the experiment. Samples taken at the indicated time points were compared with the corresponding reference sample (T0), which was taken immediately prior to S-starvation and transcript abundance in both samples was determined by microarray analysis.

**Figure 2:**
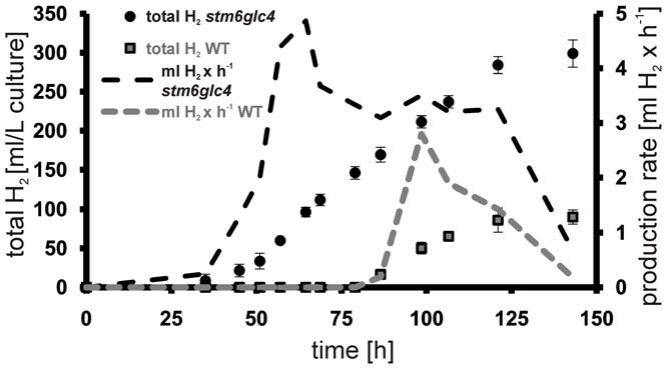
Total volume of H_2_ produced by *stm6glc4* (black circles) and WT (grey squares) during the course of the experiment. The total volume of H_2_ produced during the experiment expressed as ml/L culture is shown on the left y-axis with the duration of the experiment indicated on the x-axis. Production rates given as ml per hour are indicated on the right y-axis and presented as dotted lines.

As can be seen in Figure 1, patterns of quantum yield change differ significantly between wild type (WT) and high H_2_ production mutant *stm6glc4*. In *stm6glc4* ΦPSII declined sharply to below 0.1 within 28 h after the start of S-depletion while it decreased more slowly in the WT. Since the ΦPSII drop correlates strongly with the drop of oxygen in the culture [12], the pattern of ΦPSII changes indicates that the anaerobic phase started much earlier in *stm6glc4* than in WT (after 28 h compared to 68 h) and as a result, H_2_ production started much earlier in the mutant. This offers a significant advantage for the development of high efficiency H_2_ production processes. However, it has to be emphasized that efficient H_2_ production requires a residual activity of PSII, since the PSII-dependent H_2_ase pathway represents a vital part of the entire process [13]. Furthermore at the end of the experiment, the total amount of H_2_ produced by *stm6glc4* was over 3–5 times higher than the amount produced by the WT (Figure 2). In addition to the faster transition to anaerobiosis, peak H_2_ production rate was also higher in *stm6glc4* compared to WT (Figure 2, right y-axis; 4.9 vs. 2.8 ml•h^−1^). Within the same first 48 h of the anaerobic phase, *stm6glc4* had already produced 50% more H_2_.

In both strains, cell number increased slightly after sulfur depletion was induced, and then slowly decreased toward the end of the experiment. Surprisingly, while chlorophyll *a* to *b* ratio (Fig. 1, chl *a*/*b*) increased steadily in WT from about 2 to around 2.8, that value remained rather constant at around 2 in *stm6glc4*, indicating different adaption response to S-deprivation.

### Microarray analysis reveals numerous genes which are differentially regulated in the mutant

The detailed analysis of all microarray data including four time points in *stm6glc4* and six time points in WT led to the identification of 410 nuclear encoded genes displaying a more than 2 fold differential transcript change for at least one of the time points (T1–T4 for *stm6Glc4* and T1–T6 for WT) after sulfur deprivation in comparison to the sulfur-deplete T0 condition (151 genes in *stm6glc4* and 342 genes in WT). Among them, 189 genes could be assigned into certain functional groups while the majority of the remaining genes are not functionally annotated.

**Figure 3:**
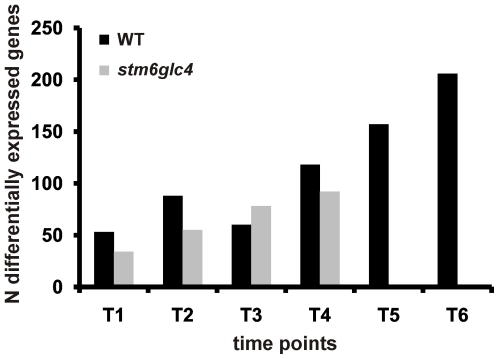
Number of differentially expressed genes identified at each time point for both strains (*stm6glc4* and WT) by microarray analyses.

**Figure 4:**
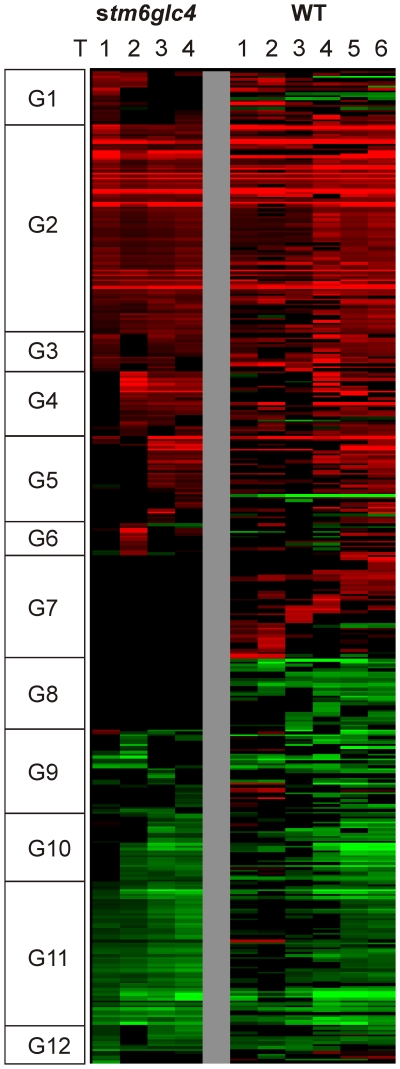
Heat map of all differentially expressed genes which were hierarchically clustered based on *stm6glc4* data set. Changes in transcript abundance compared to T0 are indicated by different colors: Red (increase), Green (decrease) and Black (unchanged). Genes were categorized into 12 different groups (G1–12) based on their expression patterns.

In both strains, the number of differentially expressed genes generally increased during the course of the experiment (Figure 3), reflecting the increasing “physiological distance” from the reference physiological state at T0. Due to two additional time points T5 and T6, the number of differentially expressed genes was significantly higher in WT, which might have been caused by a longer duration of sulfur deprivation in this case. Interestingly the number of genes differentially expressed in WT at time points T1/T2 is significantly higher than the corresponding number for *stm6glc4* at these sampling points. This points at a dampened gene regulatory response in *stm6glc4*, which could partly explain the higher susceptibility of *stm6glc4* to –S-induced photo-damage, as can be seen by the precipitous drop in photosynthetic quantum yield. To determine the time-dependent gene expression log2 ratios were first grouped by hierarchical clustering and clustered data then visualized by heat mapping (Figure 4). Based primarily on the transcript variations from *stm6glc4* data sets, 12 main expression pattern groups were identified (G1–G12). The heat map provides an overview of different expression patterns observed among the differentially expressed genes (DEGs) as well as the contrast between *stm6glc4* and WT. The list of all DEGs with their transcript abundance change at each time point is presented in Table S1.

Sulfur deprivation induced H_2_ production is a biphasic process. Within the first phase sulfur depletion impairs the PSII repair cycle and causes a declined oxygen evolving activity. Consequently oxygen evolution decreases constantly until oxygen consumption by mitochondrial respiration exceeds the rates of production [1].

The initial phase is therefore characterized by the establishment of anaerobic conditions. Once anaerobiosis has been established the H_2_ase pathway is activated. In addition to comparing the overall transcriptomic differences between WT and *stm6glc4* we were also able to distinguish between the two different stages in order to take the biphasic character of H_2_ production into account. Genes specifically up- or down-regulated in only the mutant or the WT were chosen from our array data to correlate distinctive transcriptomic changes with the phenotypical differences between WT and the mutant. Genes displaying a WT- or mutant-specific expression pattern during the establishment of anaerobiosis (Fig. 1 T1/T2) are listed in Table 1. Processes up-regulated in both strains during the first 28 h after sulfur deprivation include sulfur acquisition/recycling as well as carotenoid biosynthesis (Table 1). The up-regulation of genes encoding proteins implicated in the acquisition of sulfur is a direct consequence of sulfur depletion and has already been described [9]. An up-regulation of carotenoid biosynthesis in response to sulfur depletion has previously been demonstrated for other green algae like *Dunaliella bardawil* [23]. Although transcript abundance of all PSII associated major light-harvesting genes (*LHCBM*) except for *LHCBM9* is significantly reduced [6], the expression of stress-related *LHC* genes (Table 1; *LHCSR1/3*) is induced after withdrawal of sulfur [9], [24]. Interestingly the encoded proteins might have a higher carotenoid content compared to LHCBMs *in vivo* as suggested by refolding studies in the presence of various pigments [25].

**Table 1:** Wild Type-specific expression changes in T1 and T2.

*Upregulated*				
*Process*	*Gene/Locus*	*Description*	*ID*	*Group*
photosynthesis	*LHCSR3*/Cre08.g367400	stress-related LHC protein	8770.D	1
	*LHCSR1*/Cre08.g365900	stress-related LHC protein	251.A	7
	Cre07.g320450/Cre07.g320400	CBR-like ELIP	9621.E	7
carbon metabolism	*GWD2*/Cre07.g332300	R1 protein, α-glucan water dikinase	1457.C	9
	*GND1 a/b*/Cre12.g526800	6-phosphogluconate dehydrogenase	337.A	4
nitrogen metabolism	Cre11.g477200	NmrA-like protein	5077.C	5
	*AMT1;1*/Cre03.g159254	ammonium transporter	199.A	9
CO_2_ concentrating mechanism (CCM)	*LCR1*/Cre02.g136800	low-CO_2_ inducible Myb transcription factor	266.A	7
	*HLA3*/Cre02.g097800	C_i_ uptake	21.A	9
Amino acid metabolism:	*OAT1*/Cre11.g474800	Ornithine transaminase	6901.C	5
Lipid metabolism:	Cre01.g035350	Trans-2-enoyl-CoA reductase	1311.C	7
Nucleotide metabolism	Cre03.g184400	NUDIX_Hydrolase_19	5676.C	7
Transport:	CCP2/Cre04.g222750	putative mitochondrial carrier	128.A	7
Metabolism of cofactors:	Cre08.g359700	Lipoate synthase	2624.C	7
Vesicular transport	Cre06.g289700	TRAPP component	8048.D	7
Transcription/Translation	Cre01.g031050	SPT5 transcription elongation factor	5127.C	7
	Cre02.g102400	DNA-directed RNA polymerase SU	6049.C	7
	Cre12.g528900	PUA like RNA binding protein	3412.C	9
Proteolyis	Cre16.g663350	clp protease ATP-binding subunit	4348.C	7
Cell division	Cre12.g519700	YihA/EngB-like GTPase	7786.D	7
Downregulated				
Transcription/Translation:	Cre02.g130150	SAP-domain containing	5599.C	8
Ca^2+^ homeostasis/signaling	*ACA2*/Cre12.g505350	calcium-transporting ATPase	4472.C	8

*stm6glc4*-specific expression changes in T1 and T2				
Upregulated				
*Process*	*Gene/Locus*	*Description*	*ID*	*Group*
photosynthesis	Cre12.g505400	Chlorophyllase-like protein	9679.E	2
Lipid metabolism	Cre12.g512300	Lipoxygenase/oxylipin synthesis	7494.C	9
Transport	*CTR3*/Cre10.g434650	copper transporter	6696.C	2
Oxygen-dependent enzymes	Cre10.g443050	prolyl 4-hydroxylase	9635.E	4
	*CRD1*/Cre07.g346050	copper deficiency-/hypoxia-induced	26.A	4
Chaperones	*HSP22F*/Cre14.g617400	heat-shock response	9317.E	6
Low CO_2_-inducible genes	*LCI34*/Cre03.g191250		9652.E	4
	*LCI24*/Cre03.g179800		9154.E	6
Others	Cre13.g585550	PAP2_haloperoxidase	9401.E	2
Downregulated				
Transcription/Translation	Cre12.g498600	Elongation factor 1 alpha	9508.E	11
	Cre12.g525200	NOP56 ribosome biogenesis factor	3226.C	11

Common regulation patterns				
Upregulated				
*Process*	*Gene/Locus*	*Description*	*ID*	*Group*
PSII antenna remodelling	*LHCBM9*/Cre06.g284200	-S induced LHC protein	350.A	2
carotenoid biosynthesis				
Sulfur acquisition/recycling				
Non-phosphorylating OXPHOS by-pass	*AOX1*/Cre09.g395950	mitochondrial alternative oxidase	443.A	2
	*UCP2*/Cre06.g257550	mitochondrial uncoupling protein	5365.C	2
Downregulated				
photosynthesis				

List of genes showing either a specific down- or upregulation in one of both examined strains. Genes contained in the list displayed a 2fold down- or upregulation for at least one of the time points T1/T2. Differentially expressed genes are sorted according to the cellular processes involved as deduced from their functional annotation. Gene names are given along with the corresponding locus names (Phytozome 7.0; http://www.phytozome.net/) and a description of their function. Indicated gene IDs correspond to those given in the gal file (http://www.chlamy.org/galfile.xls/) for Chlamydomonas olinucleotide array v2.0 [50]. Heat map group assignments (Figure 4) for each gene are given as well.

Among the genes showing a differential regulation within T1/T2 were those encoding for stress-related LHC (light-harvesting proteins) proteins namely LHCSR1, LHCSR3 and a putative Cbr-like ELIP protein. LHCSR3 showed a strongly increased transcript level in the WT (12 fold in T1 vs. T0; Table S1) whereas the increase in *stm6glc4* was very moderate (2 fold in T1 vs. T0; Table S1). The physiological relevance and especially the impact of the dramatic expression induction of *LHCSR3* on H_2_ production are still unknown. LHCSR1 shows an identity of 87% to LHCSR3 but in contrast to LHCSR3, which is essential for energy-dependent quenching (qE) as demonstrated by the characterization of the knock-out mutant *npq4* [26], little is known about the physiological function of LHCSR1. The transcript of LHCSR1 is exclusively up-regulated in the WT and no expression changes upon sulfur depletion were observed for the mutant (Table S1) yielding in an about 50 fold higher transcript level in WT cells compared to *stm6glc4* cells in the phase after S-depletion and before H2 production (Figure S1; LHCSR1; preH_2_). Calcium and a plant-specific Calcium Sensor (CAS) calcium binding protein seem to be involved in the expression regulation of *LHCSR3* [27] and interestingly one of the genes differentially regulated between WT and mutant encodes a protein potentially functioning within Ca^2+^ homeostasis and signaling (*ACA2*; Table 1). In contrast to the mutant, which did not show any differential regulation of the gene *ACA2*, a significant down-regulation occurred in the WT. BLAST analyses performed with *ACA2* indicated a high homology to P_IIb_-type ATPases from *A. thaliana* (UniProtKB Q9M2L4/Q9LU41) located in the plasma membrane, vacuole, plastid envelope, or endoplasmatic reticulum and some evidence exists for the requirement of P-ATPases for proper stress-responsiveness [28].

The Cbr (carotene biosynthesis-related)-like ELIP (early light-induced protein) is a homolog (35% identity) of an ELIP-like protein identified in *Dunaliella bardawil* [29] named Cbr (UniProtKB P27516), for which zeaxanthin binding and a photoprotective role was proposed [30]. Similar to *LHCSR1* mRNA the transcript of the *Cbr-like ELIP* gene showed a considerably lower steady-state level (only 2.35±0.25% of WT level) in the mutant within T1/T2 (Figure S1; Cbr-like ELIP; preH_2_). Another transcript exclusively up-regulated in the WT encodes for a putative α-glucan water dikinase (Table 1 and Figure S1 *GWD2*; EC 2.7.9.4). GWDs phosphorylate starch at the C6 position of amylopectin-related glucosyl residues [31] and rates of starch phosphorylation were shown to be increased during net starch breakdown in *C. reinhardtii* [32]. The exclusive up-regulation of *GWD2* in the WT in response to sulfur depletion (4 fold induction T2 vs. T0; Table S1) and a 7 fold higher steady state mRNA level compared to the mutant (Figure S1) could provide an explanation for the different extents of –S-induced starch accumulation observed between WT and *stm6glc4* within T1/T2 [11]. A lower total amount of starch and a reduced net starch synthesis in the WT could be due to a higher activity of a starch phosphorylating enzyme leading to higher breakdown rates thus decreasing net synthesis. Import of glucose supplied in the media in the case of *stm6glc4* has to be considered as a contributing factor for the higher starch accumulation in the mutant. However, increased starch accumulation could also be noted for the parental strain *stm6*, which is not equipped with a hexose uptake system, if it was grown in acetate-containing TAP media [33].

We also compared the transcriptome of H_2_ producing (Fig. 1: T3/4 *stm6glc4* and T5/6 WT) cells from WT and mutant strain like it was conducted for the T1/T2 phase (Table 2). In contrast to LHCSR3 the other two stress-related light-harvesting proteins LHCSR1 and Cbr-like ELIP which were also exclusively up-regulated in the WT during T1/2 still showed a significant up-regulation compared to T0 during H_2_ production (Table 2 and Figure S1; LHCSR1 (H_2_) and CBR-like ELIP (H_2_)). The P_IIb_-type ATPase ACA2 which was down-regulated in the WT during T1/T2 was also expressed at a reduced level while cells were producing H_2_ (Table 2; *ACA2*). Expression of *ACA1* was up-regulated in the WT in T5/6 whereas no differential gene expression could be detected in the mutant within this phase (Table 2; *ACA1*). ACA1 like ACA2 displays similarity to *A. thaliana* P_IIb_-type ATPases (UniProtKB: Q9SZR1; Q9M2L4). Among the genes up-regulated (2.9 fold T6 vs T0; Table S1) in the WT but not differentially expressed in the mutant was a gene similar (20.1% identity) to *CGDL15* (Gene ID: 5718701; UniProtKB A8IWH9), which encodes for a protein harbouring a lipase 3 domain (Table 2; Cre03.g155250). The mRNA steady-state level of this gene in the WT was about 3 fold higher than in the mutant under H_2_ producing conditions (Figure S1). Class 3 lipases are triacylglycerol lipases (EC 3.1.1.3) and interestingly the exclusive up-regulation of the putative lipase correlates well with a reduction of the lipid content during H_2_ production in WT cells and with an unchanged level in the *stm6glc4* as determined by Nile red staining [11].

**Table 2:** Wild Type-specific expression changes in T5 and T6.

*Upregulated*				
*Process*	*Gene/Locus*	*Description*	*ID*	*Group*
Photosynthesis	*LHCSR1*/Cre08.g365900	stress-related LHC protein	251.A	7
	Cre07.g320450	CBR-like ELIP	9621.E	7
Carbon metabolism	Cre16.g692800	Aldo-keto reductase	698.C	7
	Cre17.g726700	putative acetate-CoA ligase	3329.C	7
Amino acid metabolism:	*HPD2*/Cre02.g136100	4-hydroxyphenylpyruvate dioxygenase	9.A	7
Lipid metabolism:	Cre01.g035350	Trans-2-enoyl-CoA reductase	1311.C	7
	Cre03.g155250	similar to *CGLD15* (TAG lipase-like)	3844.C	12
Transport:	*ZIP6*/Cre06.g299600	zinc-iron transporter	2440.C	7
Signalling:	Cre12.g520000	Ankyrin-repeat containing protein	1171.C	1
Calcium homeostasis/signalling:	*ACA1*/Cre09.g388850	calmodulin binding calcium transporting ATPase	9169.E	1
Transcription/Translation	Cre01.g031050	SPT5 transcription elongation factor	5127.C	7
	Cre04.g226400	Histone-like transcription factor	9318.E	7
	Cre06.g273900	Histone 2A	3023.C	7
Protein folding	Cre13.g603950	peptidyl-prolyl cis-trans isomerase	2173.C	7
Proteolyis	*RSE2*/Cre01.g056650	intramembrane metalloprotease	2786.C	7
Cell division	Cre12.g519700	YihA/EngB-like GTPase	7786.D	7
	*HSP22F*/Cre14.g617400	heat-shock response	9317.E	6
Sulfur acquisition/recycling	*SIR1*/Cre08.g365700	ferredoxin-sulfite reductase	8577.D 3620.C	4
Downregulated				
CO_2_ concentrating mechanism (CCM):	*CAH2*/Cre04.g223050	carbonic anhydrase, α type, periplasmic	38.A	8
	*LCR1*/Cre02.g136800	low-CO_2_ inducible Myb transcription factor	266.A	7
	*CAH4*/Cre05.g248400	mitochondrial carbonic anhydrase	91.A	9
Transcription/Translation:	Cre02.g130150	SAP-domain containing	5599.C	8
	Cre12.g504700	Histone H2B	360.A	1
	Cre06.g273900	Histone H2A	9199.E	8
	*RPL15*/Cre02.g091100	Ribosomal protein L15	9459.E	8
Ca^2+^ homeostasis/signaling	*ACA2*/Cre12.g505350	calcium-transporting ATPase	4472.C	8
Chaperones	*LCI15*/Cre16.g685050	putative metallochaperon	285.A	9
Vesicular transport	Cre06.g289700	TRAPP component	8048.D	7
	Cre06.g290100	SNARE protein	476.A	1
Signalling	*CYG54*/Cre12.g489900	Adenylate/guanylate cyclase	4565.C	8
	*AGG2*/Cre17.g738000	phototactic protein	9625.E	8
Photosynthesis	*LHCA5*/Cre10.g425900	PS I light harvesting protein	213.A	8
	*CTH1B*/Cre12.g510050	Copper target 1 protein MgPME cyclase	19.A	1
Cell wall	*PHC12*/Cre11.g472250	cell wall protein pherophorin C12	2719.C	8
	*GP2*/Cre06.g258800	Hydroxyproline-rich glycoprotein	14.A	8
Carbon metabolism	*LCI9*/Cre02.g130700	putative α-amylase	6737.C	8
Lipid metabolism	Cre03.g164350	putative Lysophospholipase II	7018.C	9
	Cre12.g512300	Lipoxygenase/oxylipin synthesis	7494.C	9
Others	Cre18.g745350	CAP10-like	5866.C	11

*stm6glc4*-specific expression changes in T3 and T4				
Upregulated				
*Process*	*Gene/Locus*	*Description*	*ID*	*Group*
Signalling	Cre02.g093850	Leucine-rich repeat containing	2315.C	4
Downregulated				
Transcription/Translation	Cre05.g233800	Glycyl tRNA synthetase	1362.C	10
	Cre12.g525200	NOP56 ribosome biogenesis factor	3226.C	11

Common regulation patterns				
Upregulated				
*Process*	*Gene/Locus*	*Description*	*ID*	*Group*
O_2_-dependent enzymes				
S acquisition/recycling				
Non-phosphorylating OXPHOS by-pass	*AOX1*/Cre09.g395950	mitochondrial alternative oxidase	443.A	2
	*UCP2*/Cre06.g257550	mitochondrial uncoupling protein	5365.C	2
**Downregulated**				
Photosynthesis				
Mitorespiration				
Translation				

List of genes showing either a specific down- or upregulation in one of both examined strains. Genes contained in the list displayed a 2fold down- or upregulation for at least one of the time points T3/T4 in *stm6glc4* or T5/T6 in the WT. Differentially expressed genes are sorted according to the cellular processes involved as deduced from their functional annotation. Gene names are given along with the corresponding locus names (Phytozome 7.0; http://www.phytozome.net/) and a description of their function. Indicated gene IDs correspond to those given in the gal file (http://www.chlamy.org/galfile.xls/) for Chlamydomonas olinucleotide array v2.0 [50]. Heat map group assignments (Figure 4) for each gene are given as well.

Several reductive pathways in the plastid rely on reduced ferredoxin as an electron donor [34]. These pathways can therefore withdraw electrons from the H_2_ase pathway thus reducing the rates of H_2_ production. An interesting finding in this regard was the stronger up-regulation of a ferredoxin sulfite reductase in the WT (Table S1; Figure S1) during H_2_ production, which was not observed for the mutant (Table 2; *SIR1*). This enzyme uses reduced ferredoxin as an electron donor [35] thereby potentially competing for electrons otherwise used by the H_2_ase pathway which could partially explain the higher rates of H_2_ production in *stm6glc4*. A function of this enzyme in the absence of sulphur in the media during H_2_ producing conditions could be the reduction of sulfite generated within sulfur redistribution pathways, which can be seen as an essential survival strategy for cells deprived of sulphur [9].

### Transcriptomic differences between WT and *stm6glc4* at T0

Gene expression in *stm6glc4* and WT was also analyzed at T0 (T0 *stm6glc4* vs. T0 WT), and four genes encoding for proteins belonging to carbon metabolism showed increased transcript levels in the mutant (Table S2). The strongest difference in the T0 expression level was found for the gene *ICL1* that codes for the enzyme isocitrate lyase (EC 4.1.3.1) whose steady-state transcript level was over 30 fold (microarray data) or 2.6 fold (RT-Q-PCR, Figure S1) more abundant in the mutant. This enzyme catalyzes the formation of succinate and glyoxylate from isocitrate in the initial step of the glyoxylate cycle [36], which enables *Chlamydomonas* to grow on acetate as a sole carbon source. A stronger expression of this enzyme indicates that the mutant consumes more acetate via the glyoxylate cycle, which is in good agreement with our metabolomics data demonstrating a higher acetate consumption of the mutant during transition to anaerobiosis when oxidative phosphorylation is still operational [11]. *stm6glc4* also showed stronger expression of ascorbate peroxidase (*APX1*) under normal conditions compared to WT suggesting that the mutant might suffer more from oxidative stress, which is consistent with an earlier finding that the parental strain *stm6* displays elevated levels of lipid hydroperoxides already in standard growth light [21].

### The reduced LHCSR3 expression in mutant *stm6glc4* is closely related to the high-light sensitive phenotype

**Figure 5:**
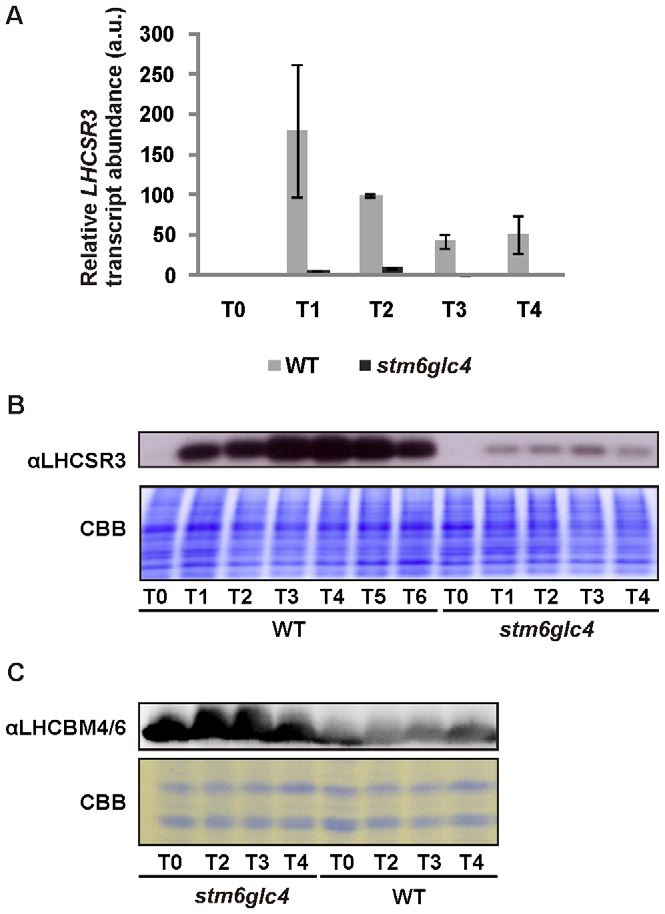
Expression of LHCSR3 in WT and *stm6glc4* under H_2_ production conditions. A: Real-time RT-Q-PCR analysis of *LHCSR3* mRNA expression in the WT (grey bars) and mutant *stm6glc4* (black bars) before (T0) and after sulphur deprivation (T1–T4). The indicated time points correspond to those used for microarray sample collection (see Fig. 1). Standard deviations are derived from measurements with three technical replicates. B: Immunoblot analysis of LHCSR3 accumulation in WT and *stm6glc4* during H_2_ production. Protein extracts were derived from the samples taken during microarray sample collection and the indicated time points correspond to those shown in Figure 1. Along with the immunodetection of LHCSR3 (αLHCSR3) a Coomassie-stained gel is shown to assess protein loading. C: Immunodetection of the major light-harvesting protein isoforms 4 and 6 (αLHCBM4/6) in protein extracts from WT and *stm6glc4*. Samples for protein extraction were taken along with the sample collection for RNA extraction prior to microarray analysis. The indicated time points are identical to the time points used for the microarray studies.

One intriguing result was the difference in the level of expression of *LHCSR3* between WT and *stm6glc4*. Consequently we decided to analyze this differential regulation in more detail. LHCSR3 has been reported to play an important role in non photochemical quenching (NPQ) [26]. The marked difference regarding the mRNA level of LHCSR3 between *stm6glc4* and WT as observed in our microarray experiments was further confirmed by real-time PCR (Fig. 5 A) and by immuno-blotting studies (Fig. 5 B; αLHCSR3). In both strains, LHCSR3 protein level increased after induction of anaerobiosis by S-starvation but the level was significantly higher in WT throughout the course of the experiment compared to that in *stm6glc4*. As suggested by the Chl *a*/*b* data (Fig. 1) recorded during H_2_ production for WT and *stm6glc4* the mutant showed a smaller LHCII antenna phenotype indicated by lower levels of the LHCBM isoforms 4 and 6 (Fig. 5 C; αLHCBM4/6) during the entire course of the experiment, suggestive of considerable differences between the stress-acclimation responses displayed by WT and mutant.

**Figure 6:**
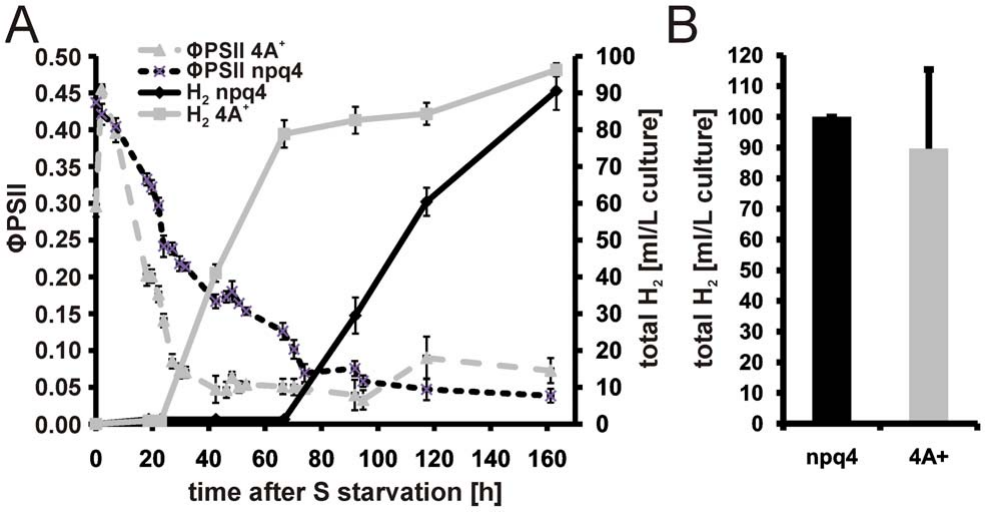
Comparison of *np4* and the WT strain 4A^+^ regarding their H_2_ production capacity and quantum yield kinetics after sulphur depletion. A: Representative H_2_ measurement with *np4* and 4A^+^. Changes in the photosynthetic quantum yield (ΦPSII) were recorded and plotted on the left y-axis. The total amount of H_2_ produced (total H_2_ [ml/L culture]) during the experiment is indicated on the right y-axis and the duration of the measurement (time after S starvation) is given on the x-axis. Black lines represent the mutant *np4* and grey lines the WT 4A^+^ (continuous line H_2_ yield; dotted line ΦPSII). Standard deviations derived from three technical replicates are indicated as error bars. B: Total H_2_ yields from *np4* (black bar) and 4A^+^ (grey bar) are given as mean values calculated from three independent biological replicates using three technical replicates per measurement. Error bars indicate standard deviations.

It was previously demonstrated that knock-out of *LHCSR3* results in a reduced fitness of *Chlamydomonas* cells grown in a changing light environment indicating a prominent role of energy-dependent quenching (qE) for cell survival under outdoor conditions [26]. Against the background that expression of LHCSR3 is rapidly induced after withdrawal of sulfur and reaches very high steady state levels it is possible that LHCSR3 accumulation under –S conditions serves to protect PSII against photodamage, especially if it is considered that an impairment of D1 *de novo* synthesis after sulfur depletion makes PSII more susceptible against light-induced damage [37]. Therefore one potential explanation for the earlier onset of H_2_ production in *stm6glc4* compared to the WT was that the lack of LHCSR3 accumulation makes PSII more prone to damage, which results in a faster transition to anaerobiosis as a precondition for an activation of the H_2_ase pathway. Consequently we investigated whether a complete loss of LHCSR3, as in the knock-out mutant *npq4* [26], [38], has any impact on the decline of PSII activity after S deprivation or on the H_2_ production capacity. The absence of LHCSR3 had no significant effect on the kinetics of ΦPSII decline (Fig. 6A) or the total H_2_ production rates (Fig. 6 B) as deduced from H_2_ experiments performed with *npq4* and WT strain (4A^+^ [26]). In conclusion the amount of LHCSR3 protein is not a decisive factor for the H_2_ production capacity under –S conditions combined with the moderate light intensity (300 µmol photons m^−2^ s^−1^) used within the present work.

**Figure 7:**
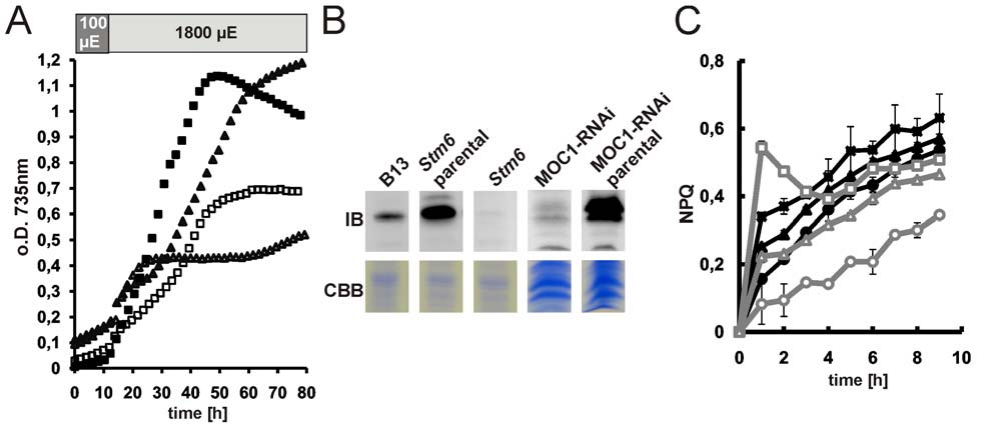
LHCSR3 expression and non-photochemical quenching capacity in *stm6*, B13, MOC1-RNAi and parental WT strains. A: Phototrophic high light growth (1800 µE) of Stm6 (open triangles), B13 (filled triangles), MOC1-RNAi (open squares) and its parental strain cc124 (filled squares) in minimal media (HSM). The light regime (15 h 100 µE; 65 h 1800 µE) is indicated with light or dark grey shaded boxes at the top of the panel. Shown is one representative growth out of three independent growth experiments. B: Immunoblot analysis (αLHCSR3) of LHCSR3 protein expression in phototrophically grown *stm6*, B13, *stm6* parental (cc1618), MOC1-RNAi and MOC1-RNAi parental (cc124) cells. Cells were first cultivated in 100 µE for 15 h before the light intensity was increased to 1800 µE. Samples for protein extraction were taken after eight hours of high-light treatment. Protein loading is shown with a Coomassie stain (CBB). C: Determination of the non-photochemical quenching (NPQ) capacity in *stm6* (circles), B13 (triangles) and *stm6* parental (squares). Cultures were either incubated in low light (filled symbols; black lines) or high light (open symbols; grey lines) prior to the measurement of NPQ over 10 min.

However, LHCSR3 has been previously shown to accumulate particularly in high light [25]–[27] and a loss of this protein causes a reduced fitness if cells are challenged by varying light intensities [26]. We consequently investigated the relationship between the genotype of *stm6* (loss of the nucleus-encoded mitochondrial protein MOC1 [21]), which is the parental strain of *stm6glc4* and the expression of LHCSR3 under high light conditions. LHCSR3 expression data obtained with the *MOC1* deletion mutant *stm6glc4* and a WT cell line after sulfur starvation (Fig. 5B) demonstrated that a loss of MOC1 protein has a profound effect on the extent of LHCSR3 accumulation, nicely emphasizing the relevance of functional inter-organelle signaling between mitochondria and chloroplasts under certain stress conditions [21]. MOC1 has been suggested to play an important role in the regulation of oxidative phosphorylation in the light and in preparing the mitochondria as a redox valve for the chloroplast thereby reducing the risk of ROS damage in particular under increasing high light conditions [21]. The knock-out of MOC1 leads to a reduced growth rate of *stm6* in high light and minimal media compared to the complemented strain *B13* (Fig. 7A). A similar growth defect could be observed for a MOC1-RNAi strain under identical conditions (Fig. 7A). Under these conditions the levels of LHCSR3 protein were almost undetectable in *stm6* and the MOC1-RNAi strain, whereas in the parental strains (cc1618 for *stm6* and cc124 for MOC1-RNAi) and in the MOC1-complemented strain B13 LHCSR3 accumulated under highlight (Fig. 7B). As was shown in a recent study, active photosynthetic electron transfer is required for proper LHCSR3 accumulation [27], so that the low amount of LHCSR3 in high-light grown *stm6* cells could indicate an impairment of photosynthetic electron transfer. In line with the finding that *stm6* fails to accumulate LHCSR3 under high light the MOC1-free mutant displayed a low NPQ capacity, if compared to B13 and its parental strain (Fig. 7C).

These results clearly demonstrate that impaired mitochondrial function affects non-photochemical quenching in the plastid and is therefore another example for the intense inter-organelle crosstalk between chloroplasts and mitochondria [39]. A correlation between impaired non-photochemical quenching and perturbed mitochondrial function has recently been reported for the CMSII mutant from tobacco, which lacks functional complex I [40]. Apart from the accumulation of key quenching proteins such as LHCSR3 cyclic electron flow (CEF) around PSI was shown to be required for normal NPQ activity [41] and interestingly one of the phenotypical characteristics of *stm6* and its derivative strains is a reduced cyclic electron flow (CEF) around PSI [33].

### MOC1 is required for functional LHCSR3 expression and MOC1 levels are inversely correlated with H_2_ production capacity

**Figure 8:**
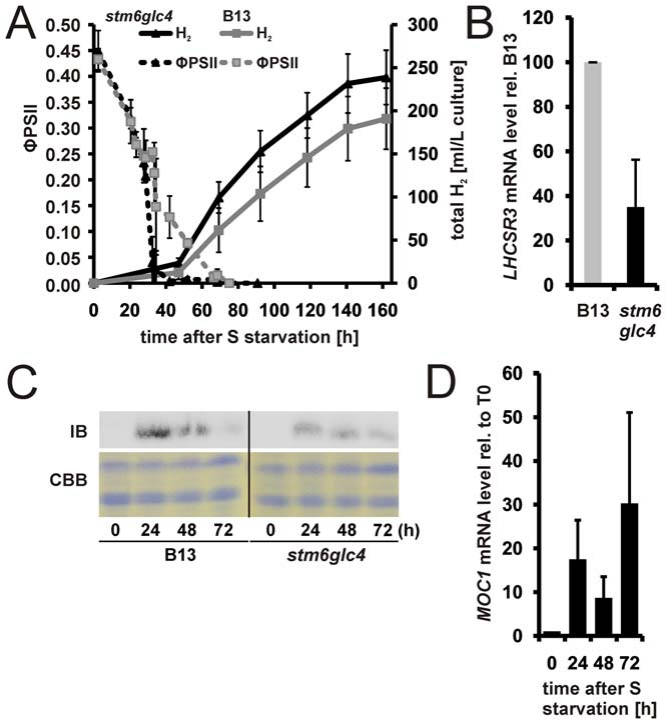
Complementation of *stm6glc4* with a functional *MOC1* gene reduces hydrogen production and restores LHCSR3 accumulation. A: Representative H_2_ measurement with B13 and *stm6glc4*. The change in the photosynthetic quantum yield (ΦPSII; left y-axis; dotted lines) was recorded along with the total amount of H_2_ produced by both cultures (total H_2_; right y-axis; continuous lines). On the x-axis the time after sulfur depletion is indicated. B13 (squares) is shown in grey and *stm6glc4* (triangles) in black. B: Abundance of *LHCSR3* mRNA after 40 hours of sulphur starvation in B13 (grey bar) and *stm6glc4* (black bar) determined by RT-Q-PCR. The mRNA level of B13 was set to 100% and standard deviations indicated by error bars represent three different experiments. C: Immunodetection of LHCSR3 in samples of B13 and *stm6glc4* after sulphur depletion with indicated duration times. The upper panel shows an anti-LHCSR3 immunoblot (IB) and the lower one a Coomassie-stained gel serving as a loading control (CBB). D: RT-Q-PCR analysis of the *MOC1* mRNA expression in WT samples taken at different time points after (24, 48 72 h) and before (0 h) sulphur depletion. Expression is given relative to T0, which was set to 1.

To correlate differences between H_2_ production in WT and *stm6glc4* with the presence or absence of functional MOC1 we also investigated the effect of *stm6* complementation on H_2_ production. The rescued cell line B13, which expresses MOC1 at WT-levels (not shown), was compared to *stm6glc4* concerning its H_2_ production capacity (Fig. 8A) and the photosynthetic quantum yield (ΦPSII) was traced during the H_2_ production experiment (Fig. 8A). Re-introduction of a functional *MOC1* gene had an adverse effect on the H_2_ production rate (Fig. 8A) clearly showing that MOC1 indeed has a strong impact on –S-induced H_2_ production. The –S- induced drop in PSII activity in B13 was almost as fast as in *stm6glc4* and both ΦPSII curves had an overall similar shape (Fig. 8A). Although the kinetics of induced PSII damage was similar between the two strains significant differences were seen in the expression levels of *LHCSR3*. Real-time data (Fig. 8 B) and immunoblot studies (Fig. 8C) showed that re-introduction of functional *MOC1* into *stm6* led to a recovery of the stress-induced expression of *LHCRS3*. However, recovery of LHCSR3 expression was not accompanied by an increased protection of PSII against photodamage under H_2_ production conditions as seen by the still rapid drop of ΦPSII in B13 and an early onset of H_2_ production comparable to that observed for *stm6glc4*. This shows again that the level of LHCSR3 accumulation has little effect on the resistance of PSII against photodamage during –S induced H_2_ production at least in moderate light. In good agreement with the hypothesis that MOC1 is deeply implicated in the acclimation processes triggered by sulfur depletion and consecutive anaerobiosis, *MOC1* mRNA levels display a steady and strong increase during H_2_ production (Fig. 8 D). In line with the finding that B13 shows a ΦPSII decline kinetics comparable to that of *stm6glc4* (Fig. 8 A), the highest mRNA level is only observed long after production of H_2_ has been initiated and not during transition to anaerobiosis.

As a conclusion our data demonstrate that the absence of MOC1 perturbs regulative mechanisms underlying the strong induction of LHCSR3 expression in response to sulfur depletion found in wild type cells. Given that the recovery of LHCSR3 expression in *B13* has little effect on the protection of PSII against –S induced photo-damage we deduce that there is no clear correlation between the available amount of LHCSR3 and the susceptibility to PSII damage under the H_2_ production conditions used in the present study.

**Figure 9:**
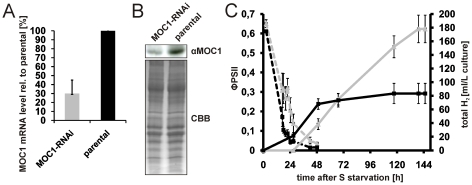
Effects on H_2_ production caused by amiRNA-mediated knock-down of *MOC1*. A: *MOC1* mRNA expression in the *MOC1* knock-down strain (MOC1-RNAi; grey bar) and the WT parental strain (parental; black bar) as determined by RT-Q-PCR. *MOC1* expression in the knock-down strain is given relative to the expression in the parental strain (set to 100%). Error bars indicate the standard deviation from three biological experiments. B: Representative immunoblot experiment (αMOC1) using an antiserum raised against MOC1 and the MOC1 knock-down strain (MOC1-RNAi) as well as its parental strain (parental). A Coomassie brilliant blue stain serves a loading control (CBB). C: Representative H_2_ measurement with the MOC1 knock-down (grey curves) and parental strain (black curves). Total H_2_ production (right y-axis; total H_2_; continuous lines) and the change of the photosynthetic quantum yield (left y-axis; ΦPSII; dotted lines) were recorded. The duration of the sulphur deplete condition is given on the x-axis. Indicated standard deviations (error bars) are derived from three technical replicates per strain. D: Result from four independent H_2_ experiments with the MOC1 knock-down strain (MOC1-RNAi; grey bar) and parental strain (parental; black bar). The H_2_ capacity (total volume of H_2_ produced) is calculated relative to the parental strain which was set to 100%. Error bars represent the standard deviation from four biological experiments with three technical replicates per experiment.

As suggested by differences seen regarding the H_2_ production capacity of *B13* and mutant *stm6glc4* and underlined by the considerable increase of *MOC1* mRNA content during H_2_ production it can be concluded that the available amount of MOC1 has a strong impact on H_2_ production. We therefore decided to prove this hypothesis by application of amiRNA-mediated knock-down of MOC1. The *pChlamyRNA3int* construct [42] was used to knock-down the expression of MOC1 in the WT strain cc124. The transformant showing the largest reduction of MOC1 expression on the mRNA (≈70±15%; Fig. 9A) and protein level (≈52±13% (n = 3); Fig. 9B) was chosen for further analysis. H_2_ production in the RNAi strain was significantly increased by ≈125% (Fig. 9C; total H_2_; continuous grey line) compared to the parental strain (continuous black line) without displaying any differences regarding the rate of PSII activity decline in response to sulfur depletion (Fig. 9C, ΦPSII MOC1-RNAi (dotted grey line) vs. parental (dotted black line)). We therefore conclude that a reduced amount of MOC1 improves H_2_ production by exerting its effects at later stages of H_2_ production.

### Summary

**Figure 10:**
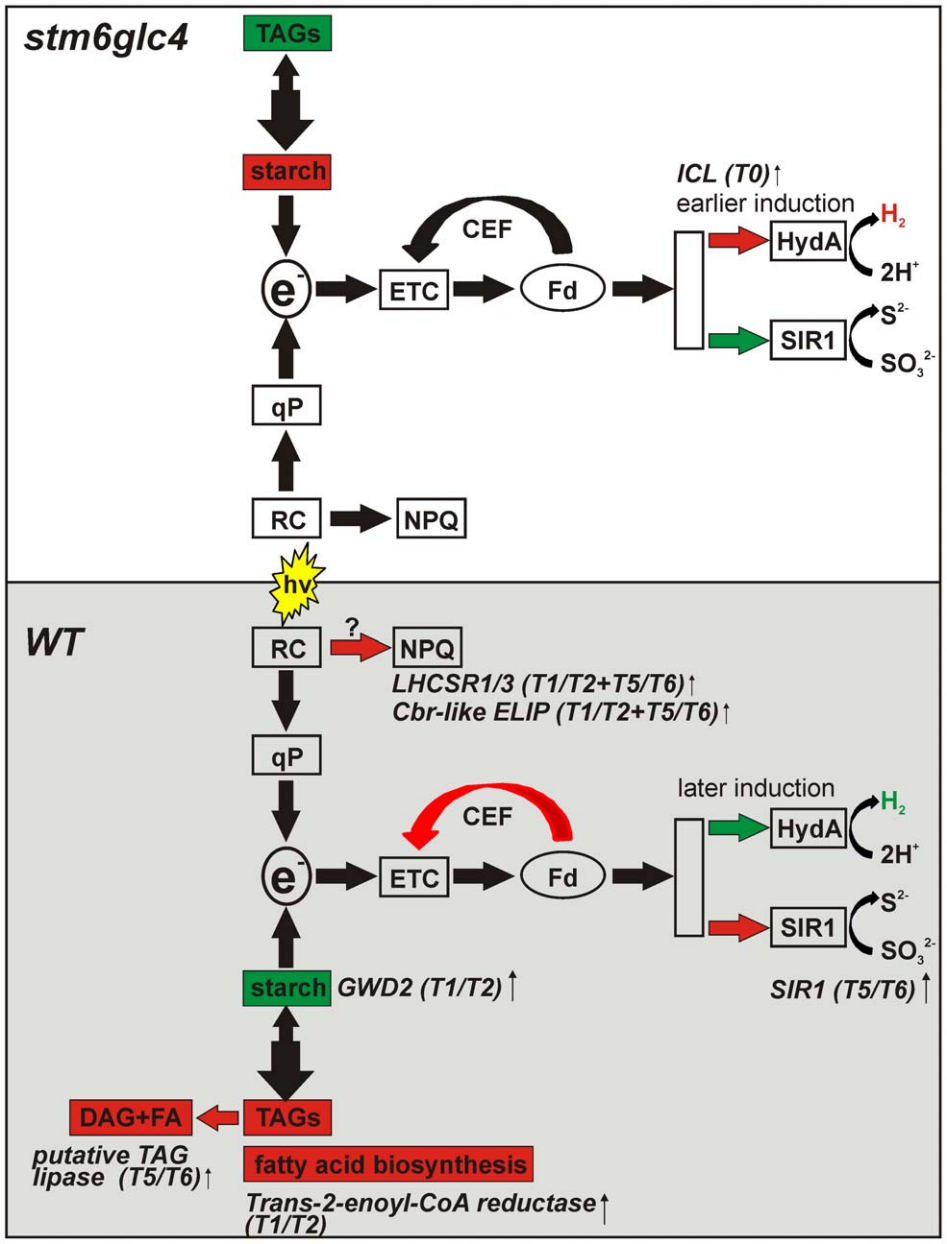
Summary of the transcriptomic differences between WT (grey shaded box) and mutant *stm6glc4* (white box) which contribute to an increased H_2_ production capacity of the mutant. A relative increase in the size of metabolite pools is indicated by a red and a decrease by a green colour. The metabolomic data used in the model were taken from Doebbe et al. [11]. The upregulation of physiological processes or pathways is also indicated by red and a downregulation by a green colour. The names of differentially expressed genes (see Table 1 and 2) are given along with the time points for which a WT- or mutant-specific regulation was identified. Abbreviations/symbols: TAGs (triacylglycerides); e^−^ (electrons fed into the H_2_ase pathway); ETC (electron transport chain); CEF (cyclic electron flow around PSI); Fd (ferredoxin); HydA (H_2_ase isoform A); SIR1 (ferredoxin-dependent sulfite reductase); qP (photochemical quenching); RC (reaction center of PSII); NPQ (non-photochemical quenching); DAG (diacylglycerol); FA (fatty acid).

In the present study, the transcriptome of a mutant displaying a high H_2_ production capacity was compared to a WT strain in order to determine which differences in expression patterns may contribute to an earlier onset, and higher rate of H_2_ production in the mutant *stm6glc4* (Figure 10). Informative differences in the expression of certain genes between mutant and WT could already be detected at T0 before the cells were resuspended in sulfur deplete media. The expression of *ICL1*, required for efficient acetate catabolism was strongly increased in the mutant providing a potential explanation for the higher acetate consumption of the mutant while anaerobiosis is being established [11]. An increased acetate consumption could be accompanied by higher rates of respiration caused by a better provision of reducing equivalents, and indeed the dissolved oxygen concentration in mixotrophic *stm6* cultures is lower than in wildtype strains [33], which can be explained by a combination of higher cyanide-insensitive respiration [21] and a lower number of active PSII complexes [33] in the mutant. Higher rates of respiration caused shorten the aerobic phase [12], [14] and indeed *stm6glc4* starts producing H_2_ much earlier than the WT strain.

*Chlamydomonas* cells accumulate energy storage compounds during the aerobic –S phase preceding the onset of H_2_ production. The most prominent compounds produced are starch and triacylglycerides (TAGs). TAGs and starch are synthesized by competing biosynthetic pathways [43] and their relevance for photobiological H_2_ production differs greatly. In contrast to starch, TAGs are not likely to be a substrate for H_2_ production, due to the impaired β-oxidation of fatty acids in an anaerobic environment, such as the conditions during H_2_ production. Starch degradation could be used to generate reducing equivalents for the non-photochemical reduction of the plastoquinon pool, via the plastidic NAD(P)H–PQ oxidoreductase Nda-2 [44], although the actual significance of this PSII-independent pathway for H_2_ production is still a matter of debate [13].

WT and mutant strains showed strong differences in their preference for one of the major energy storage compounds. The WT accumulated more TAGs than the mutant, whereas the mutant synthesized more starch and less TAGs [11]. In line with the increased TAG content in the WT the amount fatty acids produced during the aerobic phase was much higher in the WT than in the mutant [11]. An increased content of C18 fatty acids in the WT [11] during the aerobic and anaerobic phase is in good agreement with the up-regulation of a trans-2-enoyl-CoA-reductase in T1/T2 (Figure S1, *T2ECR*, preH_2_) and T5/T6, since this enzyme is needed for the elongation of fatty acids [45]. During the H_2_ production phase the WT uses more TAGs than the mutant and a putative TAG lipase showing stronger expression in the WT might be involved in this process. The lower accumulation of starch in WT cells compared to those of the mutant could be caused by a higher expression of a glucan water-dikinase (GWD2) an enzyme implicated in the breakdown of starch [31]–[32].

The stress-related LHC protein LHCSR3 was much stronger induced in the WT than in *stm6glc4*. This protein has already been shown to be required for energy-dependent quenching so that protection of PSII under high-light conditions represents a potential function of this protein. However, the increased susceptibility of PSII to –S induced photodamage, and the resulting shortened aerobic phase in the mutant, which accumulates a significantly lower amount of LHCSR3, cannot be explained by the inability to accumulate LHCSR3 amounts found in the WT. Under the moderate light conditions used in this study a complete loss of the protein, which is the case for mutant strain *npq4*, does not result in a more rapid transition from aerobic to anaerobic conditions. Furthermore no increase in the rate of H_2_ production in the anaerobic phase could be observed for the knock out mutant indicating either that energy dissipation by non-photochemical quenching (qE) does not reduce the electron flow through the H_2_ase pathway, or that qE is not triggered under the experimental conditions used in this study. High energy state quenching (qE) requires a low luminal pH [46] and biochemical analysis of LHCSR3 indicated that protonation of several residues in LHCSR3 is a precondition for active quenching [25]. The moderate light intensity used in our H_2_ experiments could have been insufficient to form a ΔpH enabling high energy state quenching. Apart from LHCSR3 the major light-harvesting protein LHCBM1 was demonstrated to represent an essential component of the non-photochemical quenching mechanism in *C. reinhartii* [47]. The expression of LHCBM1 is constantly and strongly down-regulated during the entire course of the –S induced H_2_ production process [6], so that a critical factor is almost absent although LHCSR3 is expressed at high amounts. Nevertheless it cannot be ruled out that the level of LHCSR3 present in the cell has an impact on H_2_ production at higher light intensities. Under phototrophic high light conditions in sulfur replete media a clear correlation between the presence of MOC1 and the ability to induce LHCSR3 expression could be noted (Fig. 9B). Strains expressing a low amount or no MOC1 at all showed impaired growth under high light (Fig. 9A), which was accompanied by a lack of LHCSR3 induction (Fig. 9B) and a reduced NPQ capacity (Fig. 9C).

Several reductive reactions in the plastid rely on the use of ferredoxin thus representing pathways potentially competing with proton reduction catalyzed by H_2_ase. A stronger mRNA expression of the enzyme SIR1 could provide part of the explanation for a lower rate of H_2_ production in the WT. In a previous study it was reported that *stm6* the parental strain of *stm6glc4* used in the present work is blocked in state 1 and not capable of performing cyclic electron transport around PSI [33] causing an increased supply of substrate to the H_2_ase.

### Conclusion

Our study reveals distinctive expression patterns in WT and *stm6glc4* strains during sulfur deprivation, strongly confirming previous results showing major cellular reorganizations. We also show that beside the mostly similar responses, *stm6glc4* revealed distinctive expression patterns. A preference of starch over lipids as an energy storage compound and higher rates of acetate assimilation in the aerobic phase can be deduced from our metabolomics data [11]. Our microarray data indicated a higher expression of the gene *GWD*2 exclusively found in the WT. The encoded enzyme might be implicated in starch breakdown, thus explaining why *stm6glc4* accumulates more starch in the aerobic phase. In regard to acetate assimilation and respiration during the aerobic phase preceding H_2_ we identified a higher mRNA amount for the gene *ICL1* in the mutant at T0, just before cultures have been depleted of sulfur. Reduced cyclic electron flow [33] and less competition by ferredoxin-dependent sulfite reduction as implied by the lower expression of *SIR1* contribute to the increased H_2_ production capacity of mutant *stm6glc4*. Our study revealed potential targets for the genetic engineering of *Chlamydomonas* strains aiming at higher H_2_ production capacities. Among these targets are enzymes potentially involved in starch breakdown within the aerobic phase (GWD2; glucan water-dikinase), competing ferredoxin-dependent enzymes (SIR1) and enzymes needed for efficient acetate assimilation (ICL1).

## Materials and Methods

### Strains, growth and H_2_ production conditions

The following *Chlamydomonas reinhardtii* strains were used: WT (CC-406 cw15 mt−), *stm6* and *stm6glc4*. *Stm6* was created from CC-1618 (arg7 cw15 mt−) by transformation with pARG7.8 [21]. B13 is the MOC1 complemented strain of *stm6* and was created by transforming *stm6* with a *MOC1* carrying cosmid [21]. *stm6glc4* is a derivative strain of mutant *stm6* and was generated by transformation with a vector encoding a hexose uptake symporter *HUP1* gene from *Chlorella kessleri* [22].

For knockdown of MOC1 and generation of the MOC1-RNAi strain artificial microRNA sequences were designed using Web Micro RNA designer 3 (http://wmd3.weigelworld.org):

*forward*:5′-ctagtCCGCTCGACATTCACACAATAtctcgctgatcggcaccatgggggtggtggtgatcagcgctaTATTCTGTGAATGTCGAGCGGg-3′

*reverse*:5′ctagcCCGCTCGACATTCACAGAATAtagcgctgatcaccaccacccccatggtgccgatcagcgagaTATTGTGTGAATGTCGAGCGGa-3′.

Oligonucleotide sequences were cloned into vector pChlamiRNAi3int according to Molnar *et al.* [42] to generate vector pRNAi6.18A. 2 µg of plasmid pRNAi6.18A was absorbed onto 550 nm gold particles according to the manufacturer's instructions (Seashell technologies) and a gene gun (BIO-RAD-Model PDS-1000/He Biolistic® Particle Delivery System; Bio-Rad Laboratories) was used to transform *C. reinhardtii* strain CC-124 (*mt−* 137c) by biolistic bombardment. Transformants were selected on TAP paromomycin 10 µg ml^−1^ plates and screened for stable knock-down of MOC1 by immunblot analyses and candidates were further examined by RT-Q-PCR using *MOC1*-specific primers. Non-photochemical quenching mutant *npq4* was created by transformation of CC-425 (*arg7-8 cw15 mt+ sr-u-2-60*) with pJD67 [38]. Mutant *npq4* has been determined as having a knockout in *LHCSR3* [26] while 4A+ is a WT strain [48] used as a control in previous studies on *npq4* [26].

Culturing and H_2_ production were carried out as described previously [11]. Strains lacking a hexose uptake transporter were cultured in TAP [49] and *stm6glc4* in TAP +1 mM glucose for optimal H_2_ production [22]. For the high-light growth experiment in HSM media [49] and 2% CO_2_ a photobioreactor FMT150 from PSI (Brno, Czech Republic) was used.

### Sample collection

For strain *stm6glc4*, samples from four time points were collected at 16 h, 28 h, 52 h and 76 h after sulfur starvation (T1, T2, T3 and T4 respectively). T1 represents the oxygen consuming phase while T2 marks the beginning of the anaerobic phase. At T3 and T4, the production of H_2_ was observed. For strain WT, samples were collected at slightly different time points due to slower net oxygen consumption. The first four time points T1, T2, T3 and T4 were collected at 16 h, 28 h, 52 h and 68 h, respectively. T4 marks the start of anaerobic phase as indicated by the drop of quantum yield below 0.1. In addition, two more samples were collected at 92 h (T5) and 116 h (T6) to cover the H_2_ production phase which started later in WT (see Figure 1). The samples were compared with the T0 reference samples harvested from late stationary phase cultures of the corresponding strains before S-starvation.

### RNA preparation

Samples taken from the bioreactors were immediately centrifuged (3000× *g*, 2 minutes at room temperature). Fresh cell pellets were lysed immediately with RNA Lysis Buffer (SV Total RNA Isolation System, Promega) and RNA was isolated according to the supplied manual.

### Microarray preparation and obtaining of data

*Chlamydomonas* microarray slides version 2 [50] were obtained from Dr. Author Grossman (Stanford University, USA). A recent study confirmed the specificity of 8760 features (out of 10000) according to the recent annotation [51]. Our analysis of the feature specificity according to the new annotation (Augustus 10.2) confirmed the usability of 7120 features, and just these features were included in the analysis. Microarray analysis was carried out essentially as described previously [6]. Three biological replicates for each time point were analyzed and global lowess normalization of the raw microarray data was carried out, by using the median spot intensities [6]. For each time point, a paired two-way t-test was conducted to determine significant changes in gene expression (threshold: p>0.05, within at least 4 out of 6 replica). Raw and normalized data were deposited in the GEO database (GSE30252) and followed MIAME requirements. Hierarchical clustering for both strains was performed by centroid linkage using the log2 ratios of the differentially expressed genes. Visualization was performed on Java TreeView [52].

### Quantitative real-time RT-PCR

Quantitative real time RT-PCR analysis for all genes presented in Figure 10 was carried out as previously described [53]. Briefly, Real-time RT-Q-PCR was carried out using the SensiMix One-Step kit (Quantace) in conjunction with the DNA Engine Opticon system (Bio-Rad). For each sample the cycle threshold (C_t_) values for the reference/housekeeping gene (18S rRNA) and target genes were used to calculate the relative amount of target mRNA according to the equation *rA* = *E* ^−[Ct(target gene)−Ct(reference gene)]^ with E representing the PCR efficiency according to Rasmussen [54]. Real-time data represent the median values and standard deviations of three technical replicates for each gene and time point. The primer sequences in Table 3 were used.

**Table 3:** Sequences of the primers used for RT-Q-PCR analyses of selected genes.

Gene/Locus (Accession No)/Description	Sequence 5′→3′
*ICL1*/Cre06.g282800/Isocitrate lyase	*ICL1* for: tacaactgctcgccctcttt/rev: tgaacatgccgtagttgagc
*T2ECR*/Cre01.g035350/Trans-2-enoyl-CoA-reductase	T2ECR for: agaggcagtcatccagatcg/rev: gtcctccttgagcttgtgct
*GWD2*/Cre07.g332300/R1 protein, α-glucan water dikinase	GWD2 for: atcgagcccttcaagcacta/rev: aggatgtcgtacatggtccag
*LHCSR1*/Cre08.g365900/Stress-related LHC protein	LHCSR1 for: gccatctaccacttccagca/rev: ggctcgtagtcgtccttcag
Cre07.g320450/Cre07.g320400/Cbr-like ELIP	CBR for: aagtacgttgacggcgaaat/rev: gggagtccacgttcagagag
Cre03.g155250/similar to *CGLD15* (TAG lipase-like)	TAGlip for: aacaagcggctgtatgctg/rev: cacatgagctgcagaagca
SIR1/Cre08.g365700/ferredoxin-sulfite reductase	SIR1 for: tgcagctcatgaagttccac/rev. aggtcgtccatcaccaggta
18S rRNA/AY665726, 18S ribosomal RNA	18S rRNA for: cctgcggcttaatttgactc/rev: accggaatcaacctgacaag

### Pigment measurement

Pigments were extracted from *Chlamydomonas reinhardtii* cells in 80% (v/v) acetone. The insoluble fraction was precipitated by centrifugation (2 min, 20,000× g) before measuring chlorophyll concentration and chl *a*/*b* ratio according to Arnon [55].

### Immunoblot analysis of LHCSR3 and LHCBM4/6 accumulation

For LHCSR3 immunodetection experiments samples were either taken from H_2_ producing *stm6glc4* or WT cultures at the indicated time points after S depletion (Fig. 5) or from phototrophic growth experiments conducted in PSI bioreactors using HSM minimal media (Fig. 7). For the high-light experiment in HSM cells were first cultivated in 100 µmol photons•m^−2^•s^−1^ for 15 h before the light intensity was increased to 1800 µmol photons•m^−2^•s^−1^. Samples for protein extraction were taken after eight hours of high-light treatment. LHCBM4/6 protein amounts were determined in samples taken from H_2_ producing cultures. The antisera raised against LHCSR3 and LHCBM4/6 were a kind gift from M. Hippler (IBBP, Münster University).

### Chlorophyll fluorescence measurements

Photosynthetic quantum yield ΦPSII was measured directly on bioreactors' surface with a MINI-PAM (Waltz, Germany) saturating pulse of 3,000 µmol photons•m^−2^•s^−1^.

### Measurement of non-photochemical quenching

Non photochemical quenching was measured as described in [46].

### SDS PAGE

Protein analyses were carried out using standard procedures as described in [56].

## Supporting Information

Figure S1 **Real-time RT-Q-PCR analysis of the mRNA levels of selected genes in** ***stm6glc4*** **and WT.** RNA samples of the second microarray experiment were analyzed by RT-Q-PCR in order to determine the relative amount of selected transcripts in *stm6glc4* compared to WT (set to 100%). Three different time points according to Figure 1 were analyzed: T0 (before sulfur deprivation), preH_2_ (after sulfur depletion and before H_2_ production; T2 WT; T1 *stm6glc4*) and H_2_ (H_2_ production phase; T5 WT; T3 *stm6glc4*). Standard deviations are given as error bars and were calculated from three technical replicates. Genes: *ICL1* (isocitrate lyase; Cre06.g282800; Table S2), *T2ECR* (Trans-2-enoyl-CoA-reductase; Cre01.g035350; Table 1 and 2), *GWD2* (R1 protein, α-glucan water dikinase; Cre07.g332300; Table 1), *LHCSR1* (stress-related LHC protein, Cre08.g365900, Table 1 and 2), *Cbr-like ELIP* (Cre07.g320450/Cre07.g320400; Table 1 and 2), *TAG-lipase* (similar to *CGLD15*/putative TAG lipase; Table 2), *SIR1* (ferredoxin-sulfite reductase; Cre08.g365700; Table 2).
(TIF)

Table S1 **A complete list of all microarray data.** Expression relative to T0 is given for both strains and all examined time points. GeneID numbers correspond to those contained in the gal file (http://www.chlamy.org/galfile.xls, [50]) and heat map group assignments (Figure 4) for each gene are given as well. An increase in the mRNA abundance (Tn/T0>1) is indicated by red highlighting and a decrease (Tn/T0<1) by green highlighting. Available functional assignments (column “Annotation”) are given and were used for sorting (column “Group”).
(XLS)

Table S2 **Genes showing either a specific down- or upregulation in one of both examined strains at T0.** Differentially expressed genes are sorted according to the cellular processes involved as deduced from their functional annotation. Gene names are given along with the corresponding locus names (Phytozome 7.0; http://www.phytozome.net/) and a description of their function.
(DOCX)
