# Supplementary material for: Time-Course Global Expression Profiles of Chlamydomonas reinhardtii during Photo-Biological H2 Production
Source: PLoS One. 2011 Dec 29;6(12):e29364. doi: 10.1371/journal.pone.0029364 (PMC3248568; doi:10.1371/journal.pone.0029364)
Supplement: Table S2 — Genes showing either a specific down- or upregulation in one of both examined strains at T0. Differentially expressed genes are sorted according to the cellular processes involved as deduced from their functional annotation. Gene names are given along with the corresponding locus names (Phytozome 7.0; http://www.phytozome.net/) and a description of their function. (DOCX) [file pone.0029364.s003.docx]

Supplemental Table 2

| ***Upregulated in Stm6Glc4 at T0*** | | |
| --- | --- | --- |
| *Process* | *Gene / Locus* | *Description* |
| Carbon metabolism | *TAL1* / Cre01.g032650 | Transaldolase |
|  | *ICL1* / Cre06.g282800 | Isocitrate lyase |
|  | Cre16.g675650 | Methylmalonate-semialdehyde dehydrogenase |
| Transcription/Translation | Cre10.g447800 | TROVE domain containing |
|  | Cre13.g600300 | Serine/arginine-rich pre-mRNA splicing factor |
| ROS defense | *APX1* / Cre02.g087700 | Ascorbate peroxidase |
| Chaperones | *HSP22F* / Cre14.g617400 | heat-shock response |
| ***Downregulated in Stm6Glc4 at T0*** | | |
| Signalling | Cre06.g256300 | Putative serine/threonine protein phosphatase |
| Lipid metabolism | Cre12.g512300 | Lipoxygenase |
|  | *DES6* / Cre13.g590500 | Omega-6-fatty acid desaturase, chloroplast isoform |
| Transcription/Translation | Cre12.g512400 | RNA polymerase sigma factor 54, interaction |
| other | *LCI5* / Cre10.g436550 | Low-CO_2_-inducible protein |
|  | Cre01.g015600 | HEAT-repeat containing |
